# Large Differences in Aging Phenotype between Strains of the Short-Lived Annual Fish *Nothobranchius furzeri*

**DOI:** 10.1371/journal.pone.0003866

**Published:** 2008-12-04

**Authors:** Eva Terzibasi, Dario Riccardo Valenzano, Mauro Benedetti, Paola Roncaglia, Antonino Cattaneo, Luciano Domenici, Alessandro Cellerino

**Affiliations:** 1 Istituto di Neuroscienze del CNR, Pisa, Italy; 2 Biology of Ageing, Fritz-Lipmann Institute for Age Research, Leibniz Institute, Jena, Germany; 3 Scuola Normale Superiore, Pisa, Italy; 4 Department of Genetics, Stanford University, Stanford, California, United States of America; 5 International School of Advanced Studies, Trieste, Italy; 6 European Brain Research Institute, Rome, Italy; 7 School of Medicine, University of L'Aquila, Aquila, Italy; Ecole Normale Supérieure de Lyon, France

## Abstract

**Background:**

A laboratory inbred strain of the annual fish *Nothobranchius furzeri* shows exceptionally short life expectancy and accelerated expression of age markers. In this study, we analyze new wild-derived lines of this short-lived species.

**Methodology/Principal Findings:**

We characterized captive survival and age-related traits in F1 and F2 offspring of wild-caught *N. furzeri*. Wild-derived *N. furzeri* lines showed expression of lipofuscin and neurodegeneration at age 21 weeks. Median lifespan in the laboratory varied from to 20 to 23 weeks and maximum lifespan from 25 to 32 weeks. These data demonstrate that rapid age-dependent decline and short lifespan are natural characteristics of this species. The *N. furzeri* distribution range overlaps with gradients in altitude and aridity. Fish from more arid habitats are expected to experience a shorter survival window in the wild. We tested whether captive lines stemming from semi-arid and sub-humid habitats differ in longevity and expression of age-related traits. We detected a clear difference in age-dependent cognitive decline and a slight difference in lifespan (16% for median, 15% for maximum lifespan) between these lines. Finally, we observed shorter lifespan and accelerated expression of age-related markers in the inbred laboratory strain compared to these wild-derived lines.

**Conclusions/Significance:**

Owing to large differences in aging phenotypes in different lines, *N. furzeri* could represent a model system for studying the genetic control of life-history traits in natural populations.

## Introduction

Research into aging in vertebrates is hampered by the lifespan of available model systems and tractable laboratory species with a lifespan of less than 1 year are highly desirable [1].

Annual fishes of the genus *Nothobranchius* are a clade of teleosts found in ephemeral bodies of water that form during the monsoon season in eastern and southern Africa. All surviving adults die when the habitat dries out and their maximum natural lifespan is limited to several months, making them among the shortest-lived vertebrates [2]. As an adaptation to the seasonal disappearance of their habitat, they produce desiccation-resistant eggs that can survive for one or several years encased in the dry mud in a dormant state in which all biological processes are depressed (diapause) [3]–[5]. Owing to their short lifespan, different species of annual fishes were proposed as model systems for aging research by several groups (reviewed by Genade *et al.*, [2]).

Our studies focussed on the species *Nothobranchius furzeri*, which was originally collected in 1968 in a seasonal pan of the Gona Re Zhou (GRZ) National Park in Zimbabwe, a semi-arid area with scarce and erratic precipitation [6]. The current laboratory strain, named GRZ, directly stems from this collection and was maintained as a pure line by dedicated hobbyists. A conservative estimate of 6 months generation-time implies 80 captive generations, suggesting high levels of inbreeding (confirmed by measurement of the homozygosity of polymorphic loci by Reichwald et al., submitted).

We studied GRZ and recorded a median lifespan of 9 weeks and a maximum of 12 weeks in the laboratory. This short lifespan is coupled to fast growth and accelerated expression of age-related phenotypes [2], [7]. The lifespan of *N. furzeri* GRZ can be prolonged by decreasing the water temperature [8] and by addition of the natural compound resveratrol to food [9].

Classical evolutionary theories of aging predict that senescence evolves as a result of the decreasing force of natural selection at later ages. These theories predict that populations experiencing low mortality due to external causes evolve retarded onset of senescence [10]–[13]. Later work has questioned the generality of these theories [14]–[16] and experimental tests have provided mixed results. Increasing mortality induces evolution of rapid maturation and faster senescence in *Drosophila* [17] and a comparison of longevity in chemically protected (venomous) vertebrates as compared to non-venomous sister taxa also supports the theory that reduced predation induces evolution fo retarded senescence [18]. However, a systematic study of natural populations of the small tropical teleost guppy (*Poecilia reticulata*) originating from high- and low-predation habitats did not detect any difference in longevity or span of the reproductive period [19]. For annual fish, the duration of ephemeral pans imposes an upper limit to the natural life expectancy of different populations and species of annual fishes, as adults cannot survive desiccation of their environment. Fish inhabiting habitats with shorter duration of seasonal water (i.e., more arid) may experience a shorter window of survival than fish derived from more humid habitats.

To investigate the association of life-history traits and variables related to the duration of seasonal water, we sampled the Limpopo River drainage system in southern Mozambique in 2004. This fluvial system presents a cline of altitude and annual precipitation/evaporation on a small geographical scale and we succeeded in establishing captive populations originating from different habitats along this cline. Here, we report the genetic structure of *N. furzeri* and the life-history characteristics of these wild-derived isolates.

## Results

### Collection of wild *N. furzeri*

**Figure 1:**
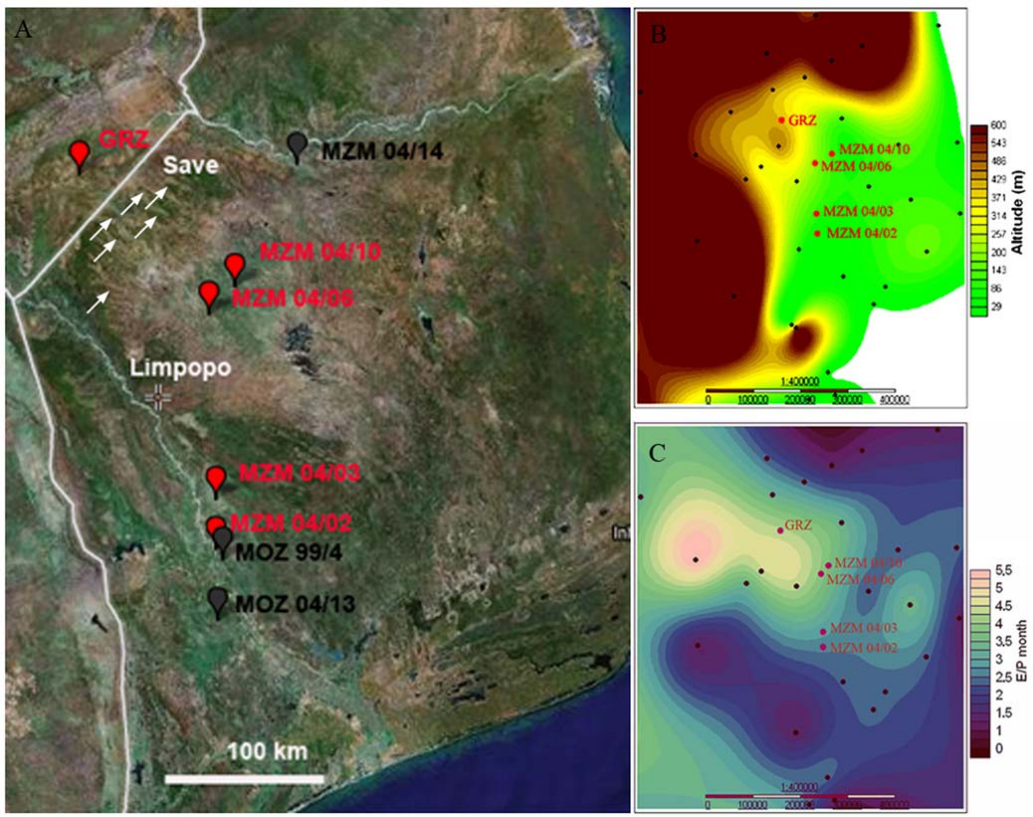
Location of *N. furzeri* habitats. (A) GPS of habitats mapped using GPS Visualizer onto a GoogleEarth image. Red flags represent habitats for lines for which lifespan data were recorded. Save and Limpopo indicate the two major rivers that limit the distribution area. Small arrows point to intermittent streams originating on the Gona Re Zhou plateau. Black flags represent habitats for which no lifespan data were recorded, but specimens were included in the phylogenetic analysis. (B) The same habitats localized onto a GIS elevation map. (C) The same habitats localized onto a GIS interpolation of the ratio between evaporation and 30-year annual average precipitation. Meteorological stations used for GIS interpolations are indicated in black.

In year 2004, we collected N.furzeri form several habitats in Mozambique, South of the original collection point in the Gona Re Zhou National Park in Zimbabwe (Fig. 1A). In particular, four populations were used in this study: MZM-04/02, MZM-04/03, MZM-04/06 and MZM-04/10 (for details on naming conventions see Methods). Localities MZM-04/02 and MZM-04/03 are close to the Limpopo River, localities MZM-04/06 and MZM-04/10 are in a system of intermittent (ephemeral) streams (Chingovo system) originating on the plateau of the Gona Re Zhou National Park in Zimbabwe. These streams do not reach the sea and disappear in a series of inland shallow lakes and swamps [6]. These two pairs of populations are therefore geographically isolated from each other.

The area sampled presents a gradient of altitude and precipitation. Altitude decreases from 400 m in GRZ to 120 m at MZM-04/10 to almost sea level at MZM-04/03 (Fig. 1B). Fig. 1C shows a GIS interpolation map of the ratio between evaporation and annual rainfall in the area of interest. An aridity cline is clearly visible. Northern populations (GRZ, MZM-04/06, MZM-04/10) originate from a typical semi-arid habitat, whereas southern populations (MZM-04/02, MZM-04/03) originate from habitats that can be considered as a transition to sub-humid. Table 1 summarizes the principal habitat characteristics for these isolates.

**Table 1:** Summary describing the isolates used in the study.

Population	Year of collection	*Elevation*	*Drainage system*	*Habitat type*
GRZ	1969	∼400 m	Chingovo	Semi-arid
MZM-04/10	2004	∼120 m	Chingovo	Semi-arid
MZM-04/06	2004	∼120 m	Chingovo	Semi-arid
MZM-04/03	2004	∼50 m	Limpopo	Transition to sub-humid
MZM-04/02	2004	∼50 m	Limpopo	Transition to sub-humid

### Genetic structure of *Nothobranchius* wild populations

A partial sequence of the mitochondrial locus *cox1* was used to define the relationship between *N. furzeri* and its sympatric/parapatric species, as well as to test for significant genetic differentiation between geographically-separated populations of *N. furzeri*. The data set included, in addition to samples collected by us, one *N. furzeri* specimen originating from a 1999 collection (Wood, 2000) and one from a 2004 collection (Brian Watters, unpublished), as well as several specimens of all known species from Mozambique and some species from coastal Tanzania.

The analysis revealed that *N. furzeri* forms a well-supported clade that also contains its sympatric species *N. orthonotus* and the parapatric species *N. kunthae*. This clade is separated from a second clade with similar distribution range that contains *N. rachovii* and other related, parapatric/allopatric species which are still undescribed. The *N.melanospilus/N.hengstleri* species complex defines a third clade and species form coastal Tanzania defines a forth clade. Deeper relationships between these clades could not be resolved and the sister clade to the *N.furzeri/N.orthonotus* species complex remains to be determined.

**Figure 2:**
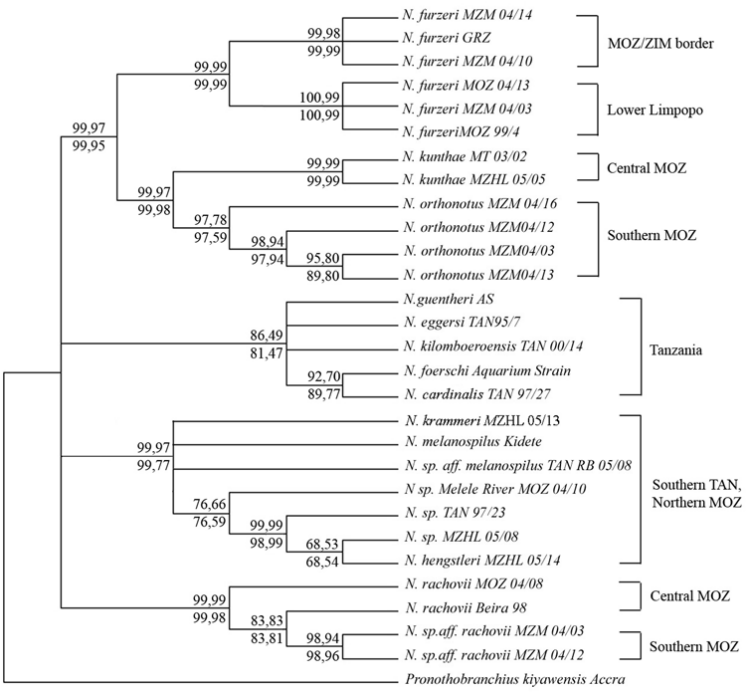
Distance-based cladogram of *Nothobranchius* from southern Africa. The cladogram is based on a partial sequence of the *cox1* mitochondrial locus. The codes after the species names refer to specific collection points. The brackets indicate the geographic origin of the clade. ZIM, Zimbabwe; MOZ, Mozambique, TAN; Tanzania. The support values over each node are the confidence probability obtained by the Minimum Evolution algorithm. The support values under each node corresponds to the Neighbor Joining algorithm. The left values corresponds to interior-branch test [40] and the right values to 3000 bootstratps. All trees used maximum composite likelihood (MCL) Computations were performed using MEGA 4.0 [38].

Populations of *N. furzeri* showed a significant level of genetic differentiation depending on their geographic origin. Three samples collected in the lower Limpopo basin clustered and were separated from three samples collected in a more northern area closer to the border between Mozambique and Zimbabwe (Fig. 2). This separation is supported by very high bootstrap values. The average sequence divergence between northern and southern populations in the analyzed region of the *cox1* locus is 4.8%. These data indicate that populations of the lower Limpopo basin are isolated from more northern populations.

### Captive lifespan of F1 and F2 generations

Individuals collected from localities MZM-04/02, MZM-04/03 and MZM-04/06 were used as founders for new wild-derived lines. Fishes collected from locality MZM-04/10 were separated into three groups and became founders of three independent lines named MZM-04/10_P_ (founders 1 male and 1 female), MZM-04/10_T_ (founders 1 male and 2 females) and MZM-04/10_G_ (founders 3 males and 5 females).

**Figure 3:**
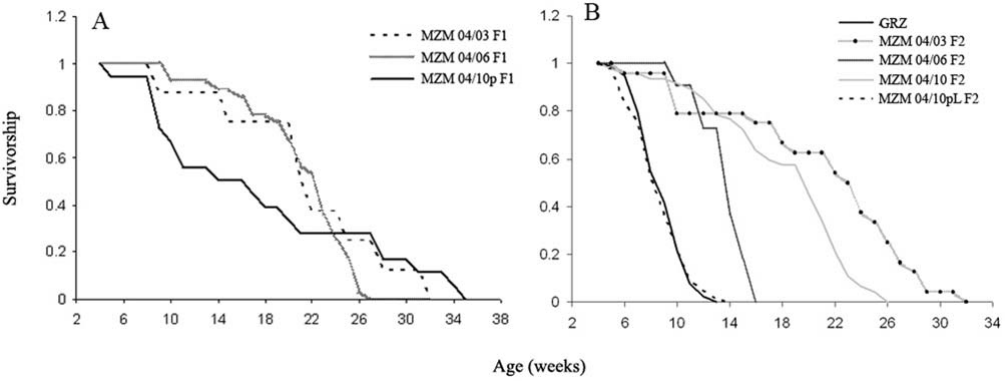
Survivorship of F1 and F2 generations. (A) Survivorship of the F1 generation of wild-derived fish MZM-04/03 (*n* = 8), MZM-04/06 (*n* = 28) and MZM-04/10_P_ (*n* = 18). (B) Survivorship of the F2 generation of MZM-04/03 (*n* = 24), MZM-04/06 (*n* = 11), MZM-04/10_G_ (*n* = 47) and MZM-04/10_P_ (*n* = 90) and of the inbred line GRZ (*n* = 93).

The survival of the F1 captive generation was recorded for MZM-04/03, MZM-04/06 and MZM-04/10_P_. The number of individuals followed over their lifespan is limited because F1 individual were intended for breeding and not for analysis of life-history traits. All isolates showed a lifespan at least double that of the laboratory strain GRZ (Fig. 3A). The lifespan of the F1 captive generation of isolates MZM-04/02, MZM-04/10_T_ and MZM-04/10_G_ could not be recorded owing to technical problems in the fish facility.

Analysis of lifespan in the F2 generation focussed on MZM-04/10 and MZM-04/03 (Fig. 3B) as representatives of northern and southern populations. Inspection of eggs after 2 months of incubation revealed the presence of developed embryos and these were then hatched. The F2 generation of MZM-04/10_P_, MZM-04/10_G_ and MZM-04/10_T_ isolates showed similar survivorship characteristics and were pooled. The median lifespan of this pooled group was 20 weeks, with 10% survivorship at 23 weeks. The median lifespan of the MZM-04/03 isolate was 23 weeks, with 10% survivorship at 29 weeks. These values were within the ranges recorded for the F1 generation of the same populations. Differences in longevity between F2 MZM-04/03 and pooled MZM-04/10 are statistically significant (log rank test, *P*<0.05).

Unexpectedly, a strong effect of incubation time on post-hatch lifespan was detected in the F2 generation of at least one isolate. Inspection of F2 eggs of the MZM-04/10_P_ isolate revealed the presence of many embryos still vital after 12 months of incubation. These were hatched in four different “cohorts” and they all showed an extremely short lifespan similar to the GRZ laboratory strain. The pooled data are reported in Fig. 3B. This isolate was named MZM-04/10_Plate_. By contrast, attempts to hatch F2 eggs of the MZM-04/03 isolate after 12-month incubation resulted in only seven vital embryos. These developed into longer-lived individuals than the late-hatched MZM-04/10_Plate_ (age at death 10–15 weeks). At this stage, the study was interrupted because the junior authors (A.C., E.T., D.V.) had to relocate for lack of funding and the fish colony was moved from SNS Pisa to FLI in Jena. Analysis of subsequent generations performed in Jena revealed that the extremely short-lived phenotype observed in the F2 generation of MZM-04/10_Plate_ is not genetically fixed (Supplementary figure S3).

A small number (*n* = 11) of F2 MZM-04/06 individuals were also followed. The data are reported in Fig. 3B. A discrepancy in the lifespan of F1 and F2 generations was observed for this isolate as well. This isolate proved difficult to breed and was lost as a consequence of the relocation from CNR in Pisa to FLI in Jena, precluding further analysis. A new collection trip is programmed to obtain new breeding stock from this locality

Maximum likelihood estimates (MLE) of the demographic parameters for three F2 isolates and the inbred laboratory stain GRZ were obtained using WinModest software. These are presented in Table 2. Differences in lifespan are accounted for by differences in the demographic rate of aging (the *b* parameter of the model). This parameter is almost six-fold higher for the GRZ strain compared to the MZM-04/03 isolate. MLE of baseline mortality (the *a* parameter of the model) showed large confidence intervals, and differences among isolates were not statistically significant.

**Table 2:** Maximum likelihood estimates of demographic parameters.

Population	Model	*a*	*b*	*s*
GRZ	Logistic	0.00012 (3.75×10^−6^−0.00365)	1.01915 (0.60581–1.71452)	1.12696 (0.31687–4.00813)
MZM-04/10_Plate_	Logistic	0.00034 (0.00003–0.00385)	0.91556 (0.59512–1.40853)	1.22287 (0.46044–3.24778)
MZM-04/10_G_	Gompertz	0.00159 (0.00049–0.00509)	0.24433 (0.18981–0.31451)	–
MZM-04/03	Gompertz	0.00275 (0.00067–0.01126)	0.17105 (0.11756–0.24889)	–

The best-fitting model was selected by comparing the likelihood of four possible models: Gompertz, Gompertz-Makeham, logistic, and logistic-Makeham. The logistic model provided the best fit of the GRZ and MZM-04/10_Plate_ data (likelihood ratio test, *P*<0.01), but not for MZM-04/10_G_ and MZM-04/03, for which the simpler Gompertz model was used. The *a* parameter represents baseline mortality, the *b* parameter represents demographic rate of aging, and the *s* parameter is the deceleration of the logistic model. Values in parentheses represent 95% confidence intervals. All simulations were performed using WinModest.

### Age-related markers: histology

**Figure 4:**
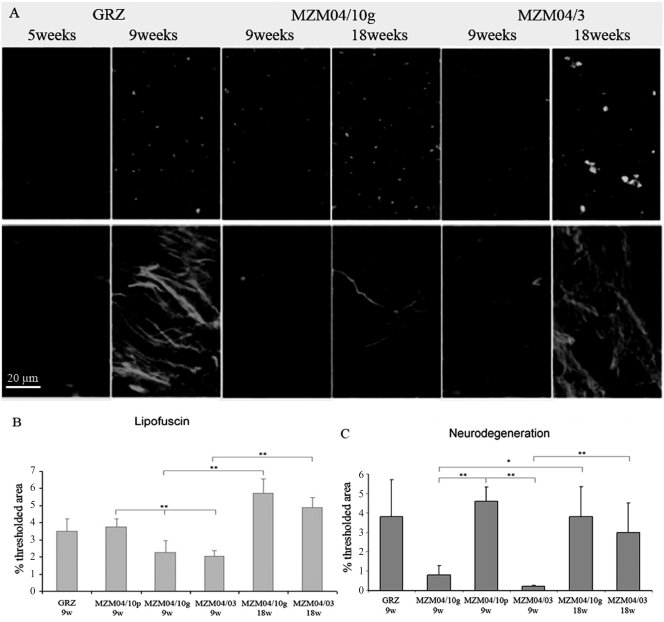
Age-dependent histological markers. (A) Representative confocal images depicting lipofuscin staining in the liver (upper row) and Fluoro-Jade B staining in the optic tectum (lower row) in the different strains at two ages. (B,C) Quantification of lipofuscin autofluorescence and Fluoro-Jade B staining as a percentage of the threshold area. Sample size *n* = 3 for all strains and ages. Error bars represent standard error of the mean. Student's *t*-test, *P<0.05, **P<0.01.

We investigated the expression of age-related histological damage in wild-derived lines and compared them with the short-lived inbred strain GRZ. Lipofuscin is an auto-fluorescent pigment that accumulates over time in a large variety of organisms [20]. We previously reported rapid accumulation of lipofuscin in the liver of the GRZ inbred strain [2], [8]. Fluoro-Jade B is a general histochemical marker of neurodegeneration [21] which was observed during aging of the GRZ inbred strain [9]. Reduced lifespan in the GRZ strain and in the long-incubation line MZM-04/10_Plate_ was coupled to accelerated expression of age-related markers in both liver and brain compared to the F2 generation of MZM-04/10_G_ and MZM-04/3 (Fig. 4A,B).

**Figure 5:**
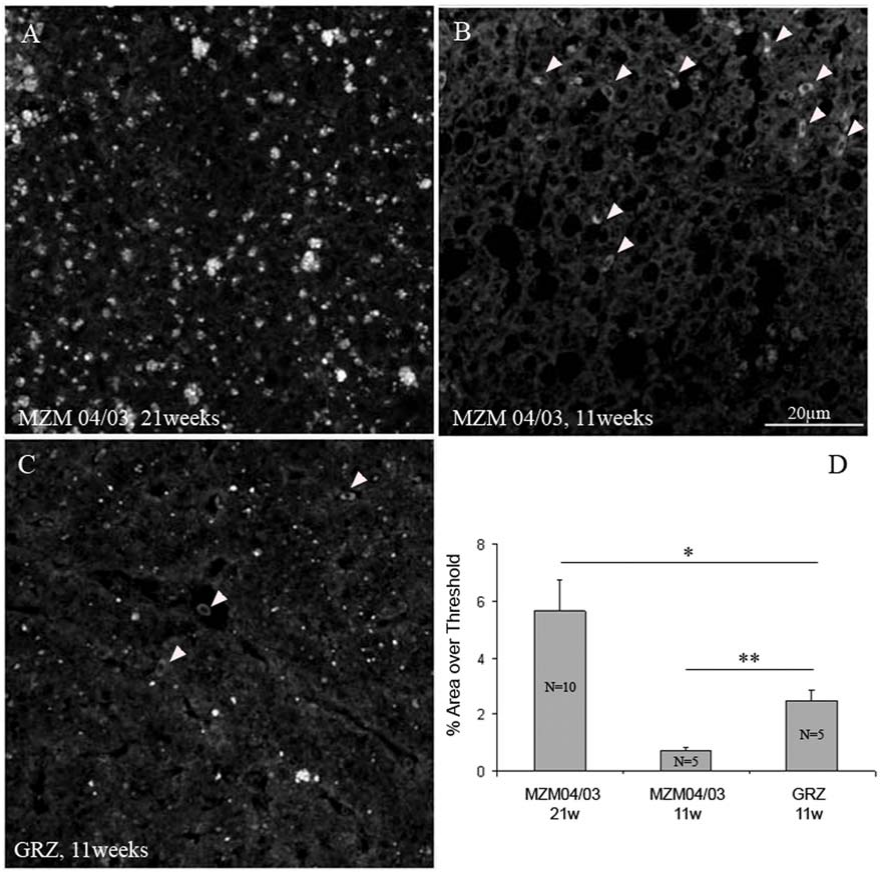
Lipofuscin in the liver. Confocal images taken at an excitation wavelength of 488 nm. Images are projections of seven confocal planes at a distance of 1 µm. (A) Liver section from 21-week-old MZM-04/03. (B) Liver section from 11-months old MZM-04/03; white arrowheads point to autofluorescent erythrocytes, which were excluded from the analysis. (C) Liver section from 11-week-old GRZ; white arrowheads point to erythrocytes, which were excluded from the analysis. (D) Quantification of lipofuscin density based on percentage threshold area. Student's *t*-test, *P<0.05, **P<0.01.

**Figure 6:**
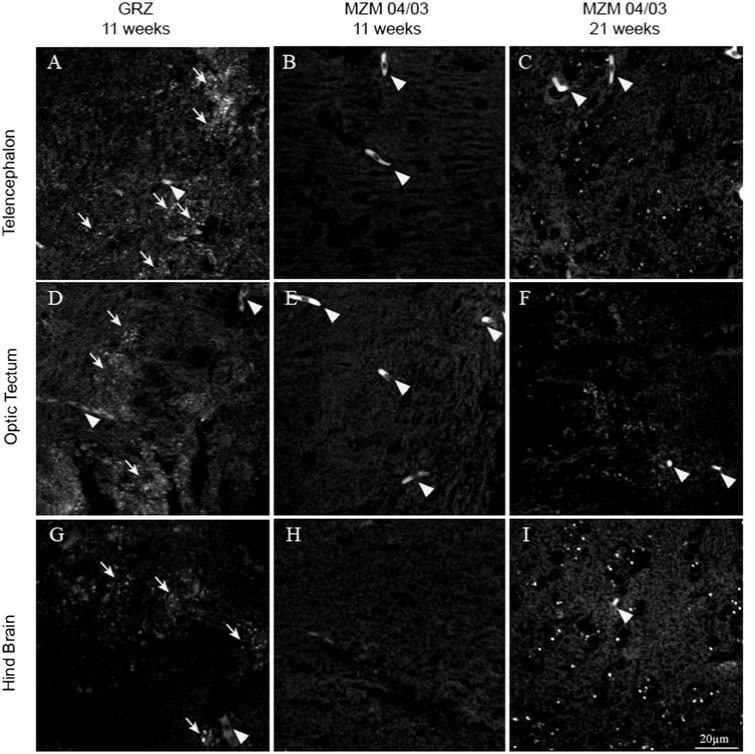
Lipofuscin in the brain. Confocal images taken at an excitation wavelength of 488 nm. Images are projections of seven confocal planes at a distance of 1 µm. (A) Telencephalon, (B) optic tectum, and (C) hindbrain from 21-week-old MZM-04/03. (D) Telencephalon, (E) optic tectum, and (F) hindbrain from 11-week-old MZM-04/03. (G) Telencephalon, (H) optic tectum, and (I) hindbrain from 11-week-old GRZ. White arrows denote lipofuscin granules. Arrowheads point to autofluorescent erythrocytes, which were excluded from the analysis.

**Figure 7:**
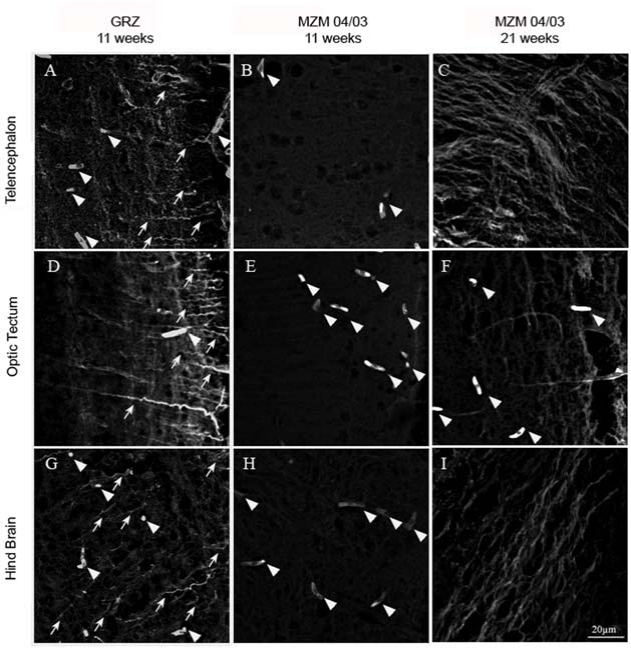
Fluoro-Jade B staining in the brain. Confocal images taken at an excitation wavelength of 488 nm. Images are projections of seven confocal planes at a distance of 1 µm. (A) Telencephalon, (B) optic tectum, and (C) hindbrain from 21-week-old MZM-04/03. (D) Telencephalon, (E) optic tectum, and (F) hindbrain from 11-week-old MZM-04/03. (G) Telencephalon, (H) optic tectum, and (I) hindbrain from 11-week-old GRZ. White arrows denote labeled neuronal processes and arrowheads point to autofluorescent erythrocytes, which were excluded from the analysis.

**Figure 8:**
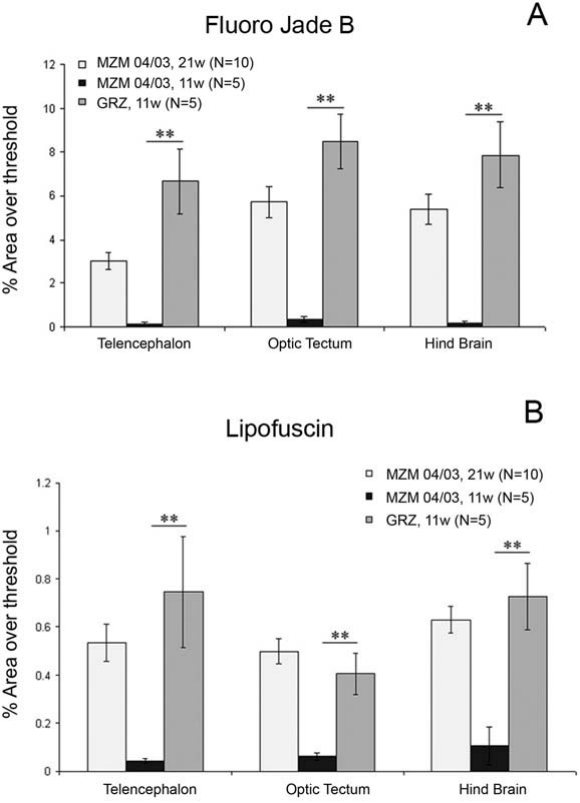
Quantification of (A) Fluoro-Jade B and (B) lipofuscin staining in the brain as a percentage of the threshold area. Data for the telencephalon, optic tectum and hindbrain are presented separately. Error bars represent the standard error of the mean. Significance is reported only for comparison between GRZ 11 weeks and MZM-04/03 11 weeks. Student's *t*-test, *P<0.05, **P<0.01.

We repeated the analysis of brain Fluoro-Jade B and liver lipofuscin in FLI in Jena after relocation on GRZ and F6 generation of MZM-04/03 using specimens bred in Jena. In addition, we analyzed expression of brain lipofuscin. Three brain areas were analyzed separately: telencephalon, optic tectum and hindbrain. Expression of liver lipofuscin was higher in the GRZ strain at 11 weeks compared to age-matched MZM-04/03 fishes, but was significantly lower than in 21-week-old MZM-04/03 (Fig. 5). Lipofuscin accumulation was also accelerated in the brain of GRZ strain (Fig. 6), a pattern paralleled by faster expression of Fluoro-Jade B positivity (Fig. 7). This acceleration affected the three brain areas equally. Quantification of both age markers in the brain revealed that 11-week-old GRZ individuals exhibited a level of expression comparable to that in 21-week-old MZM-04/03 individuals (Fig. 8).

It should be noted that several old specimens of wild-derived lines showed a macroscopic “aged” phenotype that comprises spinal curvature, emaciation and (in males) loss of color (Supplementary material, Fig. S2). This age-related phenotype is described in other fish species such as the guppy (*Poecilia reticulata*), the South American annual fish *Austrolebias bellottii* and zebrafish [22]. Although a considerable degree of variation is observed, fish from wild-derived lines may spend several weeks in this “decrepit” state before eventually dying. This is not the case for fish of the GRZ strain, which normally die before developing a macroscopic phenotype.

### Age-related markers: behavior

**Figure 9:**
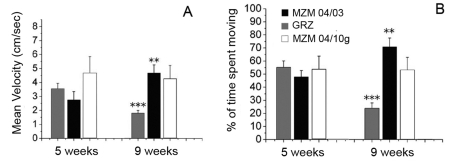
Age-dependent decrease in exploratory activity. Exploratory activity was measured in an open-field setting [9] in terms of (A) mean velocity and (B) time spent moving. Error bars represent standard error of the mean. Sample size *n* = 10 for each age and population. Pairwise comparisons were performed exclusively within populations. Mann-Whitney *U*-test, **P<0.01, ***P<0.001.

Aging in the inbred GRZ strain of *N. furzeri* is associated with reduced open-field exploration [23]. Open-field exploration was quantified at 5 weeks and 9 weeks of age in GRZ and in F2 progeny of MZM-04/10_G_ and MZM-04/3 strains. Less open-field exploration was observed only in the short-lived GRZ strain. On the other hand, the MZM-04/03 line showed an *increase* in exploratory activity in this time window. (Fig. 9)

**Figure 10:**
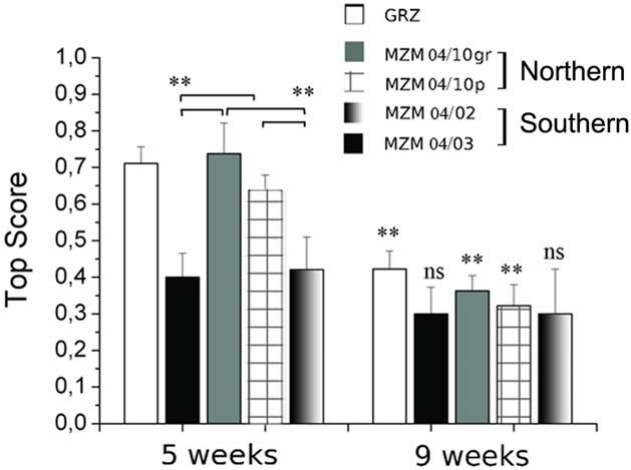
Age-dependent cognitive deficit. The average top scores of an active avoidance paradigm [9] are reported. Error bars represent standard deviation. Rank-based ANOVA, **P<0.01; ns, not significant. Comparisons *among* strains at age 5 weeks are indicated by brackets. The symbols on the 9-weeks bars refer to comparison between the two ages *within* each strain. Sample size is 10 animals for each age and strain.

Age-dependent impairment of learning and memory is a hallmark of aging in complex organisms and is observed in many model systems, including *Drosophila* [24]. Age-dependent cognitive decline was also recently described in zebrafish [25]. A form of age-dependent learning decline is observed in the GRZ strain between 5 and 9 weeks of age [23] and can be detected using a protocol of active avoidance in a modified version of the shuttle box test. We quantified age-dependent learning impairment in F2 progeny of MZM-04/10_Plate_, MZM-04/10_G_, MZM-04/3 and MZM-04/02 isolates. MZM-04/10_Plate_ and MZM-04/10_G_ showed a marked decrease in learning performance between 5 and 9 weeks of age that is reminiscent of the decline described in the GRZ strain. The MZM-04/02 and MZM-04/03 lines showed lower performance at 5 weeks of age compared to MZM-04/10_Plate_ and MZM-04/10_G_. However, their learning performance did not decrease further at 9 weeks of age (Fig. 10). Therefore, the age-dependent decline in conditioning did not correlate with captive longevity, but with the geographic origin of the animals tested.

## Discussion

We characterized longevity and age-associated histological and behavioral phenotypes in the F2 offspring of wild-derived *N. furzeri* collected by the authors in 2004. In addition, we compared the aging phenotype of captive lines derived from two habitats differing in aridity and found that age-related cognitive deficit was influenced by the geographic origin of the captive lines.

We measured the longevity of F1 and F2 offspring of fishes collected from three different habitats differing in altitude and evaporation/precipitation ratio. These out-bred populations showed median longevity of 20–23 weeks and maximum longevity of 25–32 weeks. These data, in combination with analysis of histological age markers, demonstrate that wild-derived outbred individuals of *N. furzeri* exhibit fast age-dependent decay and a short lifespan. Rapid aging is therefore a natural trait for this species.

Previous studies of the highly homozygous GRZ strain reported a median lifespan of 9 weeks and a maximum lifespan of 13 weeks. The large difference in lifespan between GRZ and wild-derived lines is certainly accounted for in part by inbreeding depression, as analysis of polymorphic markers has demonstrated that the GRZ strain is extremely homozygote (Reichwald *et al.*, submitted). However, other factors related to the captive history of the strain and its original habitat may contribute to this accelerated aging phenotype.

In mice [26] and *Drosophila* [27]–[29], involuntary selection in captivity for high productivity under benign conditions leads to rapid evolution of a shorter lifespan, an effect that is distinct from inbreeding depression. Such captive selection might have influenced the GRZ strain as well.

This study was designed to test the hypothesis that ecological variables associated with the duration of water and population isolation in the natural habitat of *N. furzeri* lead to evolution of different aging rates and longevity between fish strains derived from different regions.

The combined differences in altitude and evaporation/precipitation ratio influence the duration of the temporary habitat, since intermittent stream are drained and water accumulates in downstream habitats after the rains. Moreover, the precipitation/evaporation ratio directly influences a meteorological variable termed “length of the growing period”, which is the number of days in the year when precipitation is higher than evaporo-transpiration. The length of the growing period is usually discussed in the context of water availability for crops, but it clearly also influences the duration of seasonal ponds.

We could indeed demonstrate large genetic differences across different lines exist, which set the basis for a QTL analysis of longevity in this species (see below). Support for the life-history hypothesis, is inconclusive at this stage, however. We plan to collect and characterize more wild-derived *N. furzeri* isolates, including animals collected in GRZ as well as in the very humid habitats North of the Save River. We also plan to study the two sympatric species *N. orthonotus* and *N.* sp. aff. *rachovii*, to test for parallel evolution of life-history traits in different species of the genus *Nothobranchius*.

It is however to report that a note on breeding *N. furzeri* GRZ dating back to 1973 [30], 4 years after collection of the founders, already described a relatively short-lived phenotype (the breeding pair showed typical hunchback at 3.5 months of age and lived for 4.5 months), suggesting that alleles conferring a short-lived phenotype were present in the founder population and were fixed early during the captive history of the GRZ strain.

Although the correlation between captive lifespan and ecological conditions of the original habitat is intriguing, evolution of the short-lived phenotype in the GRZ strain can only be elucidated by studying the offspring of fish collected from the *terra typica* in GRZ as well as *N.orthonotus*, which is found in the same habitat.

We analyzed two different age-related markers: lipofuscin in brain and liver and Fluoro-Jade B in the brain. Lipofuscin is an autofluorescent pigment that accumulates with age in many animal models and is considered a robust age marker [20], [31]. We observed that lipofuscin accumulation was faster in the short-lived GRZ strain than in the longer-lived MZM-04/03 wild-derived line. This observation demonstrates that the short lifespan of the GRZ strain is coupled to faster histological aging. However, individuals from wild-derived lines can spend several weeks in a highly degenerate “decrepit” state characterized by exaggerated spinal curvature, weight loss and extreme emaciation before death. Age markers demonstrated that the aging process is accelerated in the GRZ strain, but comparison of captive longevity might overestimate the magnitude of this difference. Direct comparison of the timing of lipofuscin accumulation also pointed to differences in aging rates across organs. Brains of 11-week-old GRZ individuals showed levels of lipofuscin accumulation and Fluoro-Jade-B staining comparable to those in 21-week-old MZM-04/03 individuals. However, livers from 21-week-old MZM-04/03 individuals exhibited significantly higher levels of lipofuscin accumulation than those from 11-weeks-old GRZ individuals. These data suggest that age-dependent degeneration in the GRZ strain is faster in the brain than in the liver.

We measured age-dependent cognitive decay in the GRZ inbred strain and in four different wild-derived lines. The lines MZM-04/10_Plate_ and MZM-04/10_G_, derived from northern habitats, showed an age-dependent pattern of decline in learning performance similar to that previously reported for the GRZ inbred strain: high performance at 5 weeks of age that decreased by 9 weeks of age. It should be noted that F2 MZM-04/10_Plate_ individuals showed a short lifespan and accelerated expression of a neurodegeneration marker, whereas the MZM-04/10_G_ line showed a maximum lifespan of 26 weeks. Learning decline was not correlated with lifespan or generalized neurodegeneration. On the other hand, the lines MZM-04/02 and MZM-04/03, geographically and genetically distinct from the MZM-04/10 populations, showed low performance at 5 weeks, but no further decline by 9 weeks of age. Therefore, we could detect a clear correlation between age-dependent learning decline and the geographic origin of the lines. The difference in this age-related trait could be a neutral event stemming from genetic separation between northern and southern populations, or an evolutionary response to different ecological conditions.

Classical evolutionary theories of aging predict that populations experiencing lower mortality due to external causes evolve retarded onset of senescence compared to populations with higher extrinsic mortality [11]–[14]. This theory has so been subjected to few experimental tests in vertebrates [18], [32], [33]. Recently, a systematic study of natural isolates of the small tropical fish guppy (*Poecilia reticulata*) experiencing high and low levels of predation detected no effect of extrinsic mortality on the evolution of longevity or reproductive lifespan, but did detect an effect on functional parameters of aging [19]. The peculiar ecology of annual fishes offers a complementary model system to study the effects of extrinsic mortality on the evolution of aging. Owing to large differences in the length of the growing period, pools in semi-arid highland habitats dry out faster than pools in humid lowland habitats. Assuming that pool duration is a proxy for the maximum window of adult survivorship in the wild, fishes in more arid habitats should experience a shorter maximum lifespan than fishes in wetter habitats. Indeed, when a small sample of different species of annual fish were compared over a broad geographic scale, an indication for a correlation between meteorological conditions of the original habitat and captive lifespan was observed [2]. Our experiment was designed to test whether habitat differences can influence the evolution of life-history traits within one species. *N. furzeri* was selected because the distribution range of this species overlaps with a cline in altitude and precipitation/evaporation. We are unable to provide a definitive conclusion, as we could not obtain wild fish from GRZ (Zimbabwe), i.e., the northern end of this cline. We plan to further characterize aging phenotypes and survival in more wild-derived *N. furzeri* isolates and in the two sympatric species *N. orthonotus* and *N.* sp. aff. *rachovii* to test for the effects of extrinsic mortality on the evolution of life-history traits in *Nothobranchius*.

A clear difference was observed in age-dependent cognitive decline between the southern MZM-4/02 and MZM-4/03 lines and the northern populations, including the GRZ strain. Northern populations exhibited higher performance at younger ages, but faster cognitive decline at later ages compared to populations from more humid habitats. These results are in line with previous studies in natural populations of guppy (*Poecilia reticulata*), for which populations subject to lower extrinsic mortality exhibited lower escape performance at young ages, but a less steep age-dependent decay [19]. Both our study and that by Reznick *et al.* (2004) suggest that extrinsic mortality influences the evolution of functional life-history traits.

Different lines of *N. furzeri* exhibited large differences in life-history traits. Genome-wide scanning for quantitative traits in *N. furzeri* could represent an alternative method for identification of loci accounting for differences in longevity and age-related phenotypes in natural populations. Genomic sequences of *N. furzeri* are now available (Reichwald et al., submitted) and are being used to derive polymorphic markers for linkage studies. Analysis of backcrosses and F2 GRZ×MZM-04/03 hybrids is expected to provide a picture of the chromosomic loci responsible for the large differences in life-history traits we described here.

## Methods

### Fish collection

A form similar to *N. furzeri* was discovered in 1999 on an amateur collection trip in the lower Limpopo River drainage system in southern Mozambique. This habitat is located approximately 300 km south of the original collection point in the GRZ National Park [34]. The form is very similar in shape to *N. furzeri* but has a red, rather than yellow tail (Supplementary material, Fig. S1).

In the last week of March 2004, we sampled ephemeral pans in southern Mozambique between the Save and Limpopo Rivers. Fish were collected using fine-meshed hand-nets. Fish were bagged one per bag and all water in the bag was changed every day. Individuals of *N. furzeri* were collected from eight habitats and individuals from four habitats were successfully transported and bred in captivity. All these habitats were pools and pans in the vicinity of rivers with a muddy substrate. We established that the form depicted by Wood [34] is a different color morph of *N. furzeri*. The two morphs are distributed along a north–south gradient (Supplementary material, Fig. S1) and co-exist with two other species of *Nothobranchius*: *N. orthonouts* and *N.* sp. aff. *rachovii* [34]. A recent and more systematic surveys has largerly confirmed this biogeographic pattern [35]. The locations of the collection points are shown in Fig. 1A. Sampling was restricted to transects corresponding to primary/secondary roads. Many more *N. furzeri* habitats certainly exist in the area, but are not easily accessible. According to the convention of *Nothobranchius* collectors, collection points were named using a code containing the name of the country, the year of collection and a progressive number indicating the order of collection points along the transect. The wild-derived populations analyzed in the present study were named after their collection points: MZM-04/02, MZM-04/03, MZM-04/06 and MZM-04/10. Localities MZM-04/02 and MZM-04/03 Habitat characteristics are reported in Table 1

### Meteorological analysis

GIS maps were computed using FieldPro v. 0.91. Precipitation, altitude and evaporation data were obtained from the website http://geonetwork3.fao.org/climpag/agroclimdb_en.php and monthly mean values are reported on graphs. Fig. 1 shows the ratio of monthly normalized evaporation to monthly normalized precipitation.

### Fish culture

Eggs were maintained on wet peat moss at room temperature in sealed Petri dishes. When embryos had developed, eggs were hatched by flushing the peat with tap water at 16–18°C. Embryos were scooped up and transferred to a clean vessel. Fry were fed with newly hatched *Artemia nauplii* for the first 2 weeks and then weaned with finely chopped *Chironomus* larvae. Fishes were starting in the fourth week of life, when they are considered sexually mature, fish were moved to 40-l tanks at a maximum density of 20 fishes per tank. Filtration was provided with air-driven sponge filters. The temperature was maintained at 25°C using stab heaters (in Pisa) or by climatising the entire room (Jena). Light/dark cycles were amntained 12∶12 using a timer. Fish were fed twice a day with frozen *Chironomus larvae* at a quantity that they consumed in 30 min. *Chrimonous larvae* in Pisa were purchased from EscheMatteo (Parma, Italy). *Chrimonous larvae* in Pisa were purchased from Poseidon Aquakultur (Ruppichterot, Germany). Twice a week the bottom of the tanks was siphoned and 50% of the water was exchanged with tempered tap water.

### Survival assay

Surviving fish were counted every week starting from the forth week. Mortality in fishes younger than 4 weeks usually occurred in the first days after hatching. Dead fish were not counted because they decay fast in water and may be eaten by their tankmates before they are noticed. To compute differences among treatments, we used commercially available GraphPad and Origin programs.

Demographic analysis was performed using WinModest software (http://www.hcoa.org/scott/softw-winmodest.asp).

### Histology and histochemistry

Fishes were euthanized with MS-222 and cooled on crushed ice for 5 min before dissection. Target tissues were dissected and fixed by immersion in 4% paraformaldehyde/0.1 M phosphate buffer (pH 7.4). Fishes analyzed in Pisa were infiltrated with 30% sucrose to ensure cryoprotection, embedded in Tissuetek (Reichart-Jung, Nubloch, Germany), frozen at −20°C, and serially sectioned (thickness 18 µm) using a cryostat. Fishes analyzed in Jena were embedded in Paraplast and sections of 5 µm in thickness were cut.

Intracellular accumulation of lipofuscin during aging was detected in brain and liver tissues from young and old fishes as light blue autofluorescent granules under UV excitation. For quantification, images were acquired using a Leica confocal microscope (in Pisa) or a Zeiss LSM (in Jena) at an excitation wavelength of 488 nm, with fixed confocal parameters (pinhole, photo-multiplier, laser intensity, etc.).

Fluoro-Jade B staining [21] was used to visualize neurodegeneration in brain tissues. Neurofibrillary tangles in brain tissues from young and old fishes were quantified by analysis of confocal images acquired using the same procedure as for lipofuscin quantification.

Fluorescence analysis of images for both lipofuscin and Fluoro-Jade B staining was performed using Metamorph software.

### Open-field-like assay

Single fish were scored for locomotor activity in a 20-l test-tank at the same temperature as the home tank. Video recordings were made using a digital video camera from above and the water level was kept very low to minimize displacement on the *z*-axis, which would not be picked up by the camera while recording from above. Fishes were allowed to habituate for 30 min within the tank before the 10-min recording was started. Image analysis was performed using Ethovision software (Noldus, Wageningen, The Netherlands) to compute the mean maximum velocity and the percentage time spent moving for each fish belonging to all experimental groups. For every group, the mean and standard deviation were computed.

### Active avoidance task

Active avoidance was measured using a modified version of the shuttle box [9]. A tank (38 cm×23 cm×18 cm) was divided in two by a hurdle with a rectangular hole (3 cm×3 cm). The two compartments were wedged-shaped to funnel a fish through the hurdle. The tank was filled with water from the housing tank and the fish was left to acclimate for 15 min before starting the test. Then the conditioned stimulus (red light) was delivered in the compartment where the fish was present, followed by an adverse stimulus. The fish always responded to the disturbance by moving to the other compartment. The aim of the test was to detect the acquisition of a strategy to escape from the adverse stimulus by crossing the hurdle upon presentation of the conditioned stimulus. The conditioned stimulus lasted for 30 s. If the fish did not move to the other compartment after 15 s, the adverse stimulus was delivered for 15 s. The fish moved to the other compartment, resting for 30 s, and then the cycle was repeated. If the fish crossed the hurdle within 15 s (i.e., before the onset of the conditioned stimulus), the trial was scored as “success”, otherwise it was scored as “failure”. A complete session consisted of 50 consecutive trials.

Two indexes were scored to assess learning in each experimental group. The first measure was the performance index, which was used to visualize the evolution of the performance as a function of the trial. Successful trials were scored as 1 and failures as 0. For each trial, we computed an average score; for example, for trial 1 in *n* fishes, the average score is:
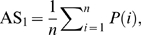
where *P*(*i*) is 0 or 1. The *performance index* (PI) was then obtained by averaging 10 consecutive AS values from trials 10 to 50 and plotting each score as follows: PI(10–50) = AS_1–10_, AS_2–11_, …, AS_41–50_. The *top score index* (TSI) was measured as the mean of the highest scores reached by individuals from one group during 50 trials. Compared to PI, TSI provides an absolute measure of ability to succeed in a task, independent of the trial. TSI is computed as the mean for all individuals in an experimental group as:
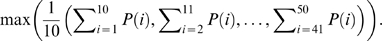



### Molecular phylogeny

Primers for amplification of *cox1* in *Nothobranchius* were those reported by [36] (Cox1for, AAC ACC TAT TCT GAT TCT TT; and Cox1rev, CAA TAA TGG CAA ATA CTG C). DNA was extracted from fin clips preserved in ethanol. PCR conditions were as follows: 5 min at 94°C; 5 cycles of 94°C for 45 s, 43°C for 30 s, and 72°C for 30 s; 25 cycles of 94°C for 45 s, 48°C for 30 s, and 72°C for 30 s; and a final 15 min at 72°C.

Amplicons were cloned in a pGEMTeasy vector and sequenced in both directions. Sequences were aligned using the program CLUSTAL W [37] followed by manual inspection and modification. Sequences have been deposited in GenBank under accession numbers EF464684–EF464713. For analysis, the primer sequences were trimmed to leave a sequence of 437 bp. Sequences were analyzed using MEGA 4.0 [38] with indels or ambiguities deleted from the analyses. T he maximum composite likelihood [39] with rate heterogeneity (Γ = 0.5) was used. We also used the Minimum Evolution algorithm [40], Log(Det) distances [41] and the Kimura 2 correction parameter [42]. All methods retrieved the same clades. Confidence probabilities [41] for branches of the NJ tree were assessed using the interior-branch test implemented in MEGA. We opted to use this method based on data suggesting that the bootstrap conservatively underestimates the statistical support for groupings within a topology, particularly when large numbers of taxa are being analyzed [43]. Trees were rooted with *Pronothobranchius kiyawense*, the species of a monotypic sister genus.

## Supporting Information

Figure S1 Distribution of N.furzeri color morphs
(0.10 MB PDF)

Figure S2 Macroscopic phenotype of senescent N.furzeri
(0.03 MB PDF)

Figure S3 Analysis of subsequent generations. The F3 generation of the MZM-04/10Plate isolate could not be analyzed because the authors (AC, ET, DV) had to relocate their laboratories and establish new fish facilities. Subsequent generations were hatched after 2–3 months of incubation, but their lifespan was not recorded as animals were sacrificed for other purposes. A survivorship analysis of MZM-04/10Plate was performed in Jena and results were compared with GRZ raised in Jena (Fig. 9). Longevity of the GRZ strain in Jena was substantially longer compared to Pisa, with a median lifespan of 11.5 weeks and 10% survivorship at 15 weeks. This difference is not unexpected given differences in water chemistry and food source between the two sites. Analysis revealed that the extremely short-lived phenotype observed in the F2 generation of MZM-04/10Plate is not genetically fixed. Median lifespan of MZM-04/10Plate in Jena was 29 weeks, with 10% survivorship at 41 weeks. A lifespan characterization of MZM-04/03 line in Jena is yet to be completed.
(0.63 MB TIF)
