# Supplementary material for: Large Differences in Aging Phenotype between Strains of the Short-Lived Annual Fish Nothobranchius furzeri
Source: PLoS One. 2008 Dec 4;3(12):e3866. doi: 10.1371/journal.pone.0003866 (PMC2585814; doi:10.1371/journal.pone.0003866)
Supplement: Figure S1 — Distribution of N.furzeri color morphs (0.10 MB PDF) [file pone.0003866.s001.pdf]

Supp. Fig. 1

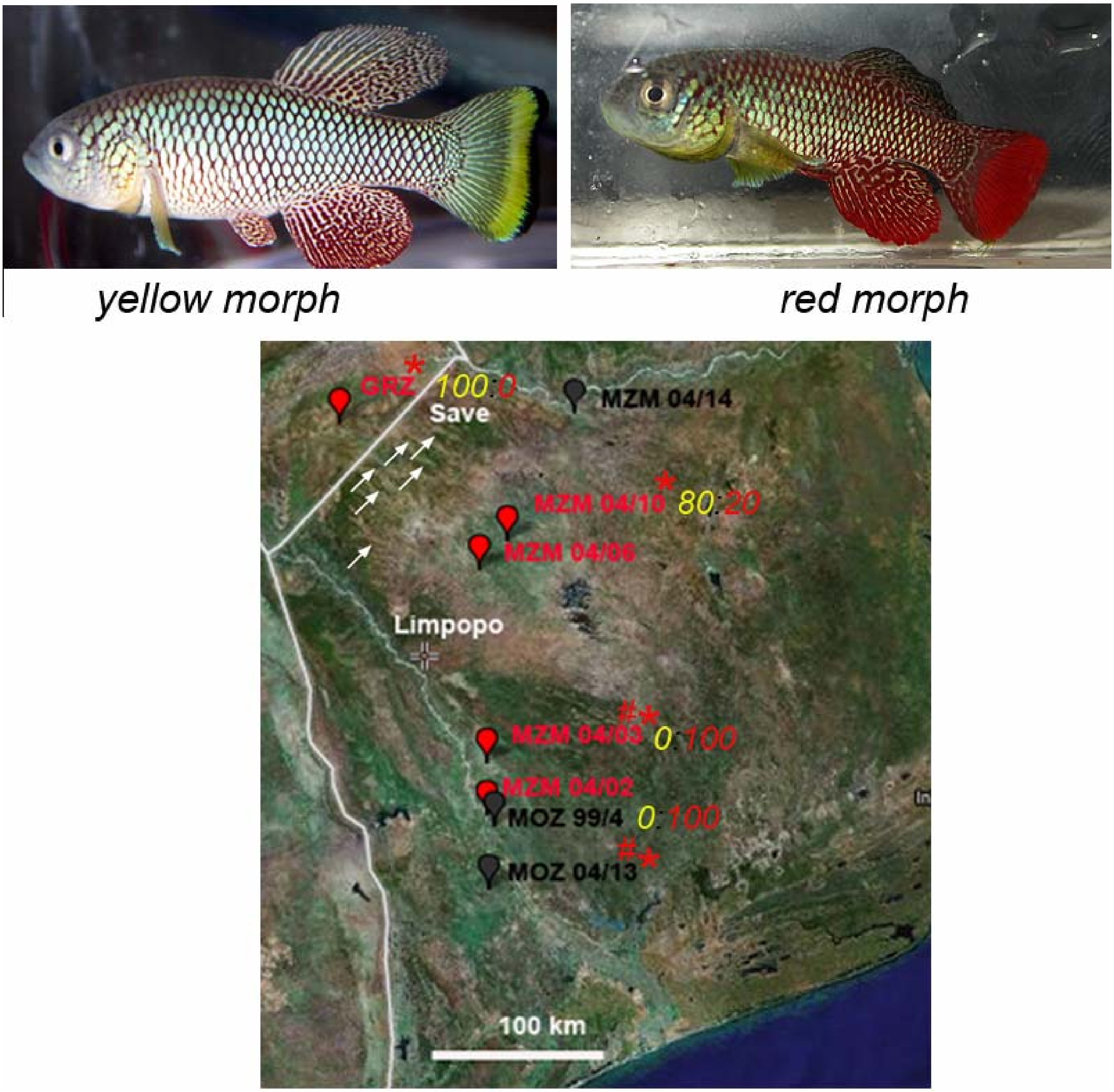

N. furzeri color morph distribution.

Top row: the two color morphs of *N.furzeri.* Central picture. The proportion of the two color morphs is shown for GRZ, MZM-04/10, MZM 04/03 and MOZ 99/4. Data for GRZ are taken from Jubb (1971) and for MOZ 99/4 from Wood (2000). * indicates localities where also *N.orthonotus* was collected and # localities where *N. sp. aff. rachovii* was collected.
