# Supplementary material for: Large Differences in Aging Phenotype between Strains of the Short-Lived Annual Fish Nothobranchius furzeri
Source: PLoS One. 2008 Dec 4;3(12):e3866. doi: 10.1371/journal.pone.0003866 (PMC2585814; doi:10.1371/journal.pone.0003866)
Supplement: Figure S2 — Macroscopic phenotype of senescent N.furzeri (0.03 MB PDF) [file pone.0003866.s002.pdf]

Supp. Fig. 2

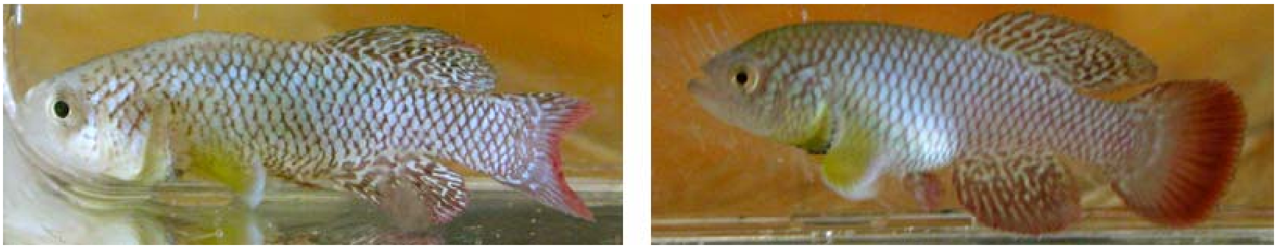

six-months old　　　　Six-weeks old

Phenotypic differences between young and old males of the MZM-04/03 line. Note emaciation and curved spine in the old fish. From Genade et al., (2005)
