# Supplementary figures and images for: Large Differences in Aging Phenotype between Strains of the Short-Lived Annual Fish Nothobranchius furzeri
Source: PLoS One. 2008 Dec 4;3(12):e3866. doi: 10.1371/journal.pone.0003866 (PMC2585814; doi:10.1371/journal.pone.0003866)

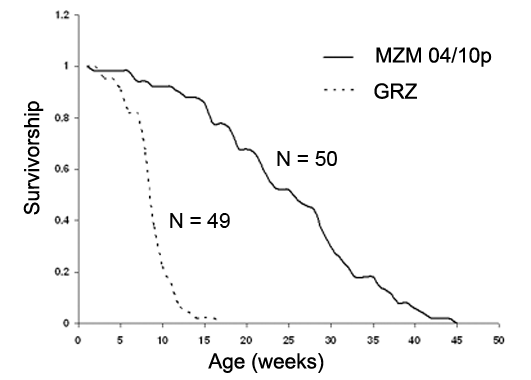

Supplement: Figure S3 — Analysis of subsequent generations. The F3 generation of the MZM-04/10Plate isolate could not be analyzed because the authors (AC, ET, DV) had to relocate their laboratories and establish new fish facilities. Subsequent generations were hatched after 2–3 months of incubation, but their lifespan was not recorded as animals were sacrificed for other purposes. A survivorship analysis of MZM-04/10Plate was performed in Jena and results were compared with GRZ raised in Jena (Fig. 9). Longevity of the GRZ strain in Jena was substantially longer compared to Pisa, with a median lifespan of 11.5 weeks and 10% survivorship at 15 weeks. This difference is not unexpected given differences in water chemistry and food source between the two sites. Analysis revealed that the extremely short-lived phenotype observed in the F2 generation of MZM-04/10Plate is not genetically fixed. Median lifespan of MZM-04/10Plate in Jena was 29 weeks, with 10% survivorship at 41 weeks. A lifespan characterization of MZM-04/03 line in Jena is yet to be completed. (0.63 MB TIF) [file pone.0003866.s003.tif]
